# Enhancing the expression of the unspecific peroxygenase in *Komagataella phaffii* through a combination strategy

**DOI:** 10.1007/s00253-024-13166-7

**Published:** 2024-05-06

**Authors:** Li-Xiang Zhao, Shu-Ping Zou, Qi Shen, Ya-Ping Xue, Yu-Guo Zheng

**Affiliations:** 1 Key Laboratory of Bioorganic Synthesis of Zhejiang Province, College of Biotechnology and Bioengineering, Zhejiang University of Technology, Hangzhou, 310014 People’s Republic of China; 2 Engineering Research Center of Bioconversion and Biopurification of Ministry of Education, Zhejiang University of Technology, Hangzhou, 310014 People’s Republic of China

**Keywords:** Copy number, Heterologous expression, High-density fermentation, Protein disulfide isomerase, Unspecific peroxygenase

## Abstract

**Abstract:**

The unspecific peroxygenase (UPO) from *Cyclocybe aegerita* (AaeUPO) can selectively oxidize C–H bonds using hydrogen peroxide as an oxygen donor without cofactors, which has drawn significant industrial attention. Many studies have made efforts to enhance the overall activity of AaeUPO expressed in *Komagataella phaffii* by employing strategies such as enzyme-directed evolution, utilizing appropriate promoters, and screening secretion peptides. Building upon these previous studies, the objective of this study was to further enhance the expression of a mutant of AaeUPO with improved activity (PaDa-I) by increasing the gene copy number, co-expressing chaperones, and optimizing culture conditions. Our results demonstrated that a strain carrying approximately three copies of expression cassettes and co-expressing the protein disulfide isomerase showed an approximately 10.7-fold increase in volumetric enzyme activity, using the 2,2′-azino-bis(3-ethylbenzothiazoline-6-sulfonic acid) as the substrate. After optimizing the culture conditions, the volumetric enzyme activity of this strain further increased by approximately 48.7%, reaching 117.3 U/mL. Additionally, the purified catalytic domain of PaDa-I displayed regioselective hydroxylation of R-2-phenoxypropionic acid. The results of this study may facilitate the industrial application of UPOs.

**Key points:**

• *The secretion of the catalytic domain of PaDa-I can be significantly enhanced through increasing gene copy numbers and co-expressing of protein disulfide isomerase.*

• *After optimizing the culture conditions, the volumetric enzyme activity can reach 117.3 U/mL, using the 2,2’-azino-bis(3-ethylbenzothiazoline-6-sulfonic acid) as the substrate.*

• *The R-2-phenoxypropionic acid can undergo the specific hydroxylation reaction catalyzed by catalytic domain of PaDa-I, resulting in the formation of R-2-(4-hydroxyphenoxy)propionic acid.*

**Supplementary information:**

The online version contains supplementary material available at 10.1007/s00253-024-13166-7.

## Introduction

**Fig. 1:**
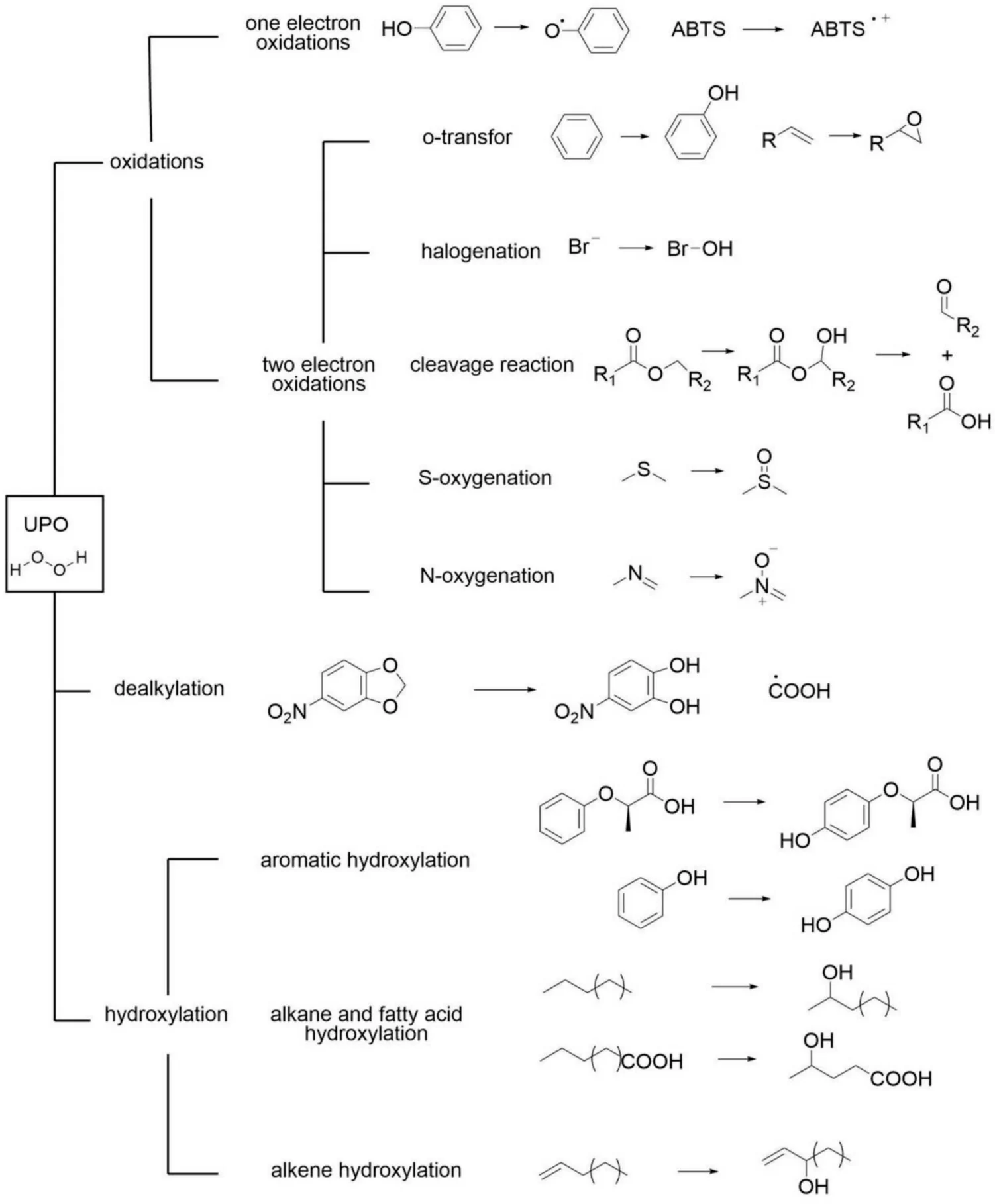
The reaction types of UPOs and examples

Biocatalysts that selectively oxidize C–H bonds have extensive prospects for industrial applications. One of the most studied classes of such biocatalysts is cytochrome P450 (P450). A typical example is the bioconversion process for pravastatin formation using *Streptomyces carbophilus* CYP105A3 (Sakaki 2012). However, the current use of P450 in the industry is still relatively limited, primarily due to its requirement for an electron transfer chain and cofactors, as well as its poor stability (Aranda et al. 2021). The unspecific peroxygenases (UPOs, E.C. 1.11.2.1) were discovered in 2004 as a novel heme-thiolate peroxidase with mono(per)oxygenase activity, which can also catalyze the selective oxidation of C–H bonds (Ullrich et al. 2004). The UPOs use hydrogen peroxide as both the primary electron acceptor and the source of oxygen (Romero et al. 2022). Additionally, they are naturally secreted into the extracellular environment in their native state and exhibit relatively high stability (Linde et al. 2022). These properties have garnered widespread attention for UPOs in the field of biocatalysis. To date, more than 300 UPO substrates have been reported (Hofrichter et al. 2015), and this number continues to grow, as depicted in Fig. 1.

The efficient expression of UPO in commonly used industrial hosts is a major challenge for its industrial application. Although there have been reports of soluble expression of UPO in *Escherichia coli*, the expression efficiencies were relatively low (Carro et al. 2019; Dolores Linde et al. 2020). Currently, most studies expressed UPO in *Saccharomyces cerevisiae* (*S. cerevisiae*) or *Komagataella phaffii* (*K. phaffii*, formerly known as *Pichia pastoris*) (Kinner et al. 2021). The main reason is likely that the maturation of UPO involves post-translational modifications such as signal peptide cleavage and glycosylation, processes that can be efficiently carried out in eukaryotic hosts like yeast. Patricia Molina-Espeja et al. achieved a groundbreaking soluble, active, and stable expression of the UPO from *Cyclocybe aegerita* (AaeUPO) in *S. cerevisiae* through directed evolution (*Cyclocybe aegerita* was formerly known as *Agrocybe aegerita*) (Molina-Espeja et al. 2014). Their evolved AaeUPO variant (PaDa-I), containing nine mutations, exhibited a volumetric enzyme activity increase of approximately 3250-fold compared to the wild-type sequence. In the PaDa-I, four mutations were located in the evolved signal peptide (S_PaDa−I_), and five were in the evolved catalytic domain (PaDa-I-CD). When the S_PaDa−I_ was used to express the wild-type mature protein sequence, the volumetric enzyme activity increased by approximately 27-fold compared with the native signal peptide of AaeUPO (S_AaeUPO_), demonstrating the importance of the signal peptide for UPO expression. The S_PaDa−I_ was subsequently successfully employed for expressing two UPOs from *Candolleomyces aberdarensis* (*C. aberdarensis*) in yeasts (Gomez de Santos et al. 2021), and the expression of these two UPOs in *K. phaffii* was more than 20 times higher than in *S. cerevisiae*. Pascal Püllmann et al. recently developed a combined promoter and signal peptide shuffling system for UPO expression in *K. phaffii* (Püllmann et al. 2021; Püllmann and Weissenborn 2021). Surprisingly, their results showed the mutual interaction between the promoter and signal peptide. They also found that the expression efficiency of PaDa-I-CD using the UPO signal peptide from *Galerina marginata* (S_Gma_) was even more than six times that of S_PaDa−I_.

The novel strategies mentioned above for enhancing UPO expression in *K. phaffii* mainly focused on optimizing elements within the expression cassette. It is widely known that methods to improve the efficiency of expressing foreign proteins in *K. phaffii* also include increasing gene copy numbers, co-expressing molecular chaperones, optimizing expression conditions, and other approaches (Fischer and Glieder 2019). It would be interesting and practical to validate the effectiveness of these strategies for PaDa-I-CD expression.

A literature has shown that AaeUPO can catalyze the biological transformation of R-2-phenoxypropionic acid (R-PPA) to R-2-(4-hydroxyphenoxy)propionic acid (R-HPPA) (Kinne et al. 2008), which is a valuable intermediate for the synthesis of herbicides. In order to assess the industrial potential of the efficiently expressed PaDa-I-CD, the present study attempted to replicate this reaction using it.

## Materials and methods

### Strains and plasmids

The *K. phaffii* strain GS115 was obtained from Invitrogen (Massy, USA) and served as the host strain for protein expression. The *Escherichia coli* (*E. coli*) DH5α (Invitrogen) was utilized for the propagation of recombinant vectors. The plasmids pPIC9 and pPICZαA (Invitrogen) were employed for expressing the target proteins. The plasmids pPIC3.5K and pAO815 (Invitrogen) were utilized for expressing the chaperones.

### Construction of expression vectors with different signal peptides

**Fig. 2:**
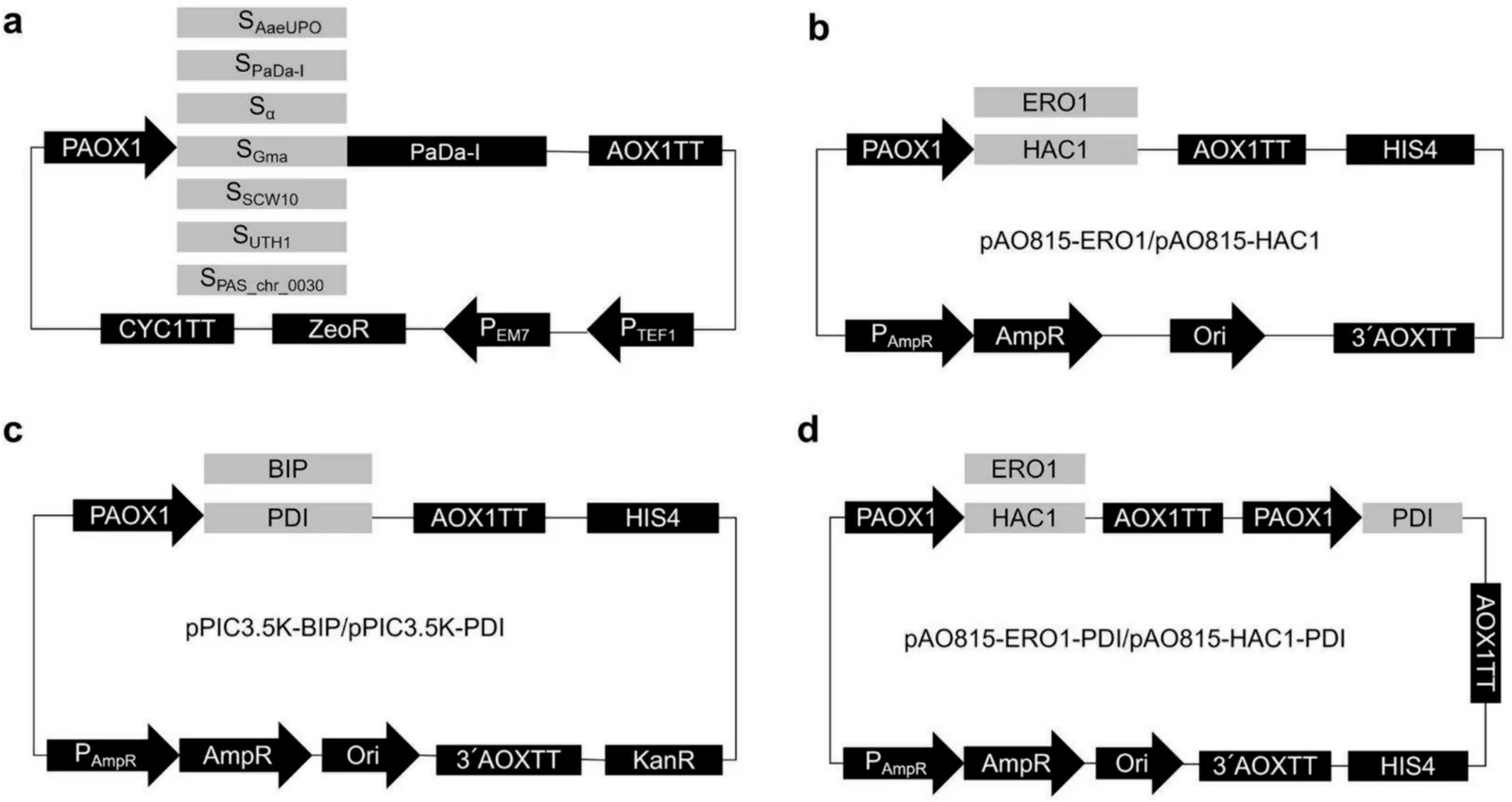
Schematic of plasmid maps. **a** Schematic of expression vectors with different signal peptide (S_AaeUPO_, S_PaDa−I_, S_α_, S_Gma_, S_SCW10_, S_UTH1_, or S_PAS_chr3_0030_). **b-d** Schematic of chaperones co-expression vectors

The gene sequence of the evolved PaDa-I containing nine mutations was synthesized and inserted into pPICZαA between the EcoR I and Not I sites by Beijing Qingke Biocompany (Beijing, China) to give pPICZ-S_PaDa−I_-PaDa-I-CD (a 6His tag was fused at its C-terminal). The DNA sequence of S_PaDa−I_ in pPICZ-S_PaDa−I_-PaDa-I-CD was mutated back to S_AaeUPO_ using site-directed mutagenesis (The Quick-change site-directed mutagenesis kit, Agilent Technologies, Santa Clara, USA) to give pPICZ-S_AaeUPO_-PaDa-I-CD with specific primers (primers used for the site-directed mutagenesis were listed in Table S1). The DNA sequence of S_Gma_ was synthesized by Beijing Qingke Biocompany. The DNA sequence of α-mating factor signal peptide (S_α_) was amplified from pPICZαA. The DNA sequences of signal peptides of SCW10 (S_SCW10_), UTH1 (S_UTH1_), and gene PAS_chr3_0030 (S_PAS_chr3_0030_) were amplified from the *K. phaffii* strain GS115 genome as described (Shen et al. 2022). Sequences of secretory peptides are listed in Table S2. The linearized pPICZ-PaDa-I-CD vector was obtained from pPICZ-S_PaDa−I_-PaDa-I-CD by reverse PCR using primers FP-pPICZ-PaDa-I-CD and RP-pPICZ-PaDa-I-CD. The DNA sequences of S_Gma_, S_α_, S_SCW10_, S_UTH1_, and S_PAS_chr3_0030_ were amplified from the synthesized product or PCR products using corresponding primer pairs and inserted into linearized pPICZ-PaDa-I-CD vector to give pPICZ-S_Gma_-PaDa-I-CD, pPICZ-S_α_-PaDa-I-CD, pPICZ-S_SCW10_-PaDa-I-CD, pPICZ-S_UTH1_-PaDa-I-CD, and pPICZ-S_PAS_chr3_0030_-PaDa-I-CD, respectively, by recombination using ClonExpress II One Step Cloning Kit (Nanjing, China) according to the manufacturer (Fig. 2a). To construct pPIC9-S_Gma_-PaDa-I-CD, the DNA sequences of S_Gma_-PaDa-I-CD was amplified from the pPICZ-S_Gma_-PaDa-I-CD using corresponding primer pairs and inserted into linearized pPIC9. All primers used for the construction of expression plasmids are listed in Table S3.

### Construction of chaperone co-expression vectors

The linearized pPIC3.5K and pAO815 vectors were obtained from pPIC3.5K and pAO815 by reverse PCR, respectively. The gene sequences of immunoglobulin-binding protein (BIP), endoplasmic reticulum oxidoreductase 1 (ERO1), transcription factor that regulates the unfolded protein response (HAC1), and protein disulfide isomerase (PDI) were amplified from the *K. phaffii* strain GS115 genome using corresponding primer pairs. The gene sequences of ERO1 and HAC1 were inserted into the linearized pAO815 to give pAO815-ERO1 and pAO815-HAC1, respectively (Fig. 2b). The gene sequences of BIP and PDI were inserted into the linearized pPIC3.5K to give pPIC3.5K-BIP and pPIC3.5K-PDI, respectively (Fig. 2c). The PDI expression cassette originating from pPIC3.5K-PDI was inserted into the linearized vectors pAO815-ERO1 and pAO815-HAC1, resulting in the development of the expression constructs pAO815-ERO-PDI and pAO815-HAC1-PDI, respectively (Fig. 2d).

### Transformation of *K. phaffii*

Plasmids constructed on the pPICZαA backbone were linearized using the restriction endonuclease Sac I and subsequently introduced into *K. phaffii* GS115 cells through electroporation. The transformed cells were plated on YPD plates (20 g/L peptone, 10 g/L yeast extract, 20 g/L glucose, and 20 g/L agar) supplemented with 100 or 800 µg/mL zeocin. The pPIC9-S_Gma_-PaDa-I-CD and plasmids constructed on the pAO815 backbone and pPIC3.5K backbone were linearized using the restriction endonucleases Sal I or Sac I. The transformed cells were plated on RDB plates (186 g/L sorbitol, 20 g/L agar, 13.4 g/L yeast nitrogen base, 20 g/L glucose, 4 × 10^−4^ g/L biotin, 0.005 g/L L-glutamic acid, 0.005 g/L L-methionine, 0.005 g/L L-lysine, 0.005 g/L L-leucine, and 0.005 g/L L-isoleucine). Positive transformants were selected by colony PCR and further confirmed by sequencing the target gene. The recombinant strains utilized in this study are listed in Table 1. Except screening multicopy strains, 25 single colonies were selected for expression level testing after each transformation. Representative strains were used for comparison in the manuscript.

**Table 1:** The recombinant strains utilized in this study

Strains	Introduced plasmids	PaDa-I copy number	Secretory peptide	Co-expressed chaperone
S_PaDa−I_-C_s_	pPICZ-S_PaDa−I_-PaDa-I-CD	1.0 ± 0.2	S_PaDa−I_	NA^1^
S_AaeUPO_-C_s_	pPICZ-S_AaeUPO_-PaDa-I-CD	1.0 ± 0.1	S_AaeUPO_	NA^1^
S_Gma_-C_s_	pPICZ-S_Gma_-PaDa-I-CD	1.0 ± 0.2	S_Gma_	NA^1^
S_α_-C_s_	pPICZ-S_α_-PaDa-I-CD	1.0 ± 0.3	S_α_	NA^1^
S_SCW10_-C_s_	pPICZ-S_SCW10_-PaDa-I-CD	1.0 ± 0.2	S_SCW10_	NA^1^
S_UTH1_-C_s_	pPICZ-S_UTH1_-PaDa-I-CD	1.0 ± 0.1	S_UTH1_	NA^1^
S_PAS_chr3_0030_-C_s_	pPICZ-S_PAS_chr3_0030_-PaDa-I-CD	1.0 ± 0.2	S_PAS_chr3_0030_	NA^1^
S_Gma_-C_s_-HIS	pPIC9-S_Gma_-PaDa-I-CD	1.0 ± 0.2	S_Gma_	NA^1^
S_Gma_-C_m1.6_	pPICZ-S_Gma_-PaDa-I-CD	1.6 ± 0.3	S_Gma_	NA^1^
S_Gma_-C_m2.7_	pPICZ-S_Gma_-PaDa-I-CD	2.7 ± 0.4	S_Gma_	NA^1^
S_Gma_-C_m4.3_	pPICZ-S_Gma_-PaDa-I-CD	4.3 ± 0.5	S_Gma_	NA^1^
S_Gma_-C_m4.5_	pPICZ-S_Gma_-PaDa-I-CD	4.5 ± 0.5	S_Gma_	NA^1^
S_Gma_-C_m2.7_-ERO1	pPICZ-S_Gma_-PaDa-I-CD pAO815-ERO1	2.7 ± 0.4	S_Gma_	ERO1
S_Gma_-C_m2.7_-HAC1	pPICZ-S_Gma_-PaDa-I-CD pAO815-HAC1	2.7 ± 0.4	S_Gma_	HAC1
S_Gma_-C_m2.7_-BIP	pPICZ-S_Gma_-PaDa-I-CD pPIC3.5K-BIP	2.7 ± 0.4	S_Gma_	BIP
S_Gma_-C_m2.7_-PDI	pPICZ-S_Gma_-PaDa-I-CD pPIC3.5K-PDI	2.7 ± 0.4	S_Gma_	PDI
S_Gma_-C_m2.7_-ERO/PDI	pPICZ-S_Gma_-PaDa-I-CD pAO815-ERO1-PDI	2.7 ± 0.4	S_Gma_	ERO1 and PDI
S_Gma_-C_m2.7_-HAC1/PDI	pPICZ-S_Gma_-PaDa-I-CD pAO815-HAC1-PDI	2.7 ± 0.4	S_Gma_	HAC1and PDI

^1^*NA* not available

### Shaking flask culture

Strains were inoculated into 5 mL of YPD medium (20 g/L peptone, 10 g/L yeast extract, and 20 g/L glucose) at 30 °C and 220 rpm until they reached an OD_600_ of 2–6. Subsequently, cells were transferred to 50 mL of BMGY medium (1 mM potassium phosphate, pH 6, 20 g/L peptone, 10 g/L yeast extract, 13.4 g/L yeast nitrogen base, 10 g/L glycerol, and 4 × 10^−4^ g/L biotin) with a 5.0% inoculum. The culture was continued in a shaker until the OD_600_ reached approximately 20. The cells were harvested by centrifugation, washed three times with pre-cooled sterile water, and then resuspended in 50 mL of fresh BMMY medium (1 mM potassium phosphate, pH 6, 20 g/L peptone, 10 g/L yeast extract, 13.4 g/L yeast nitrogen base, 10 g/L methanol, and 4 × 10^−4^ g/L biotin). Finally, the cultures were incubated at 28 °C and 220 rpm for 96 h for induced expression, with 1.0% methanol added, and samples were collected every 24 h. The protein expression levels were assessed using Sodium Dodecyl Sulfate PolyAcrylamide Gel Electrophoresis (SDS-PAGE) and Western blot analysis.

To optimize the induction temperatures, after the addition of methanol for induction, the cultures were incubated at 16 °C, 20 °C, 24 °C, or 28 °C. To optimize the induction conditions, methanol concentrations of 0.5%, 1.0%, 1.5%, and 2.0% were utilized.

### Fermentation cultivation

Strains were inoculated into 3-mL YPD tubes and incubated for 20 h at 30 °C and 220 rpm in a shaker. These cultures were then used to inoculate 50-mL YPD shaking flasks with a 10.0% inoculum and incubate at 30 °C and 220 rpm until the OD_600_ reached approximately 16. The seed culture was subsequently transferred to a 5-L fermenter with a 10.0% inoculum, and batch fermentation was conducted using BSM medium (26.7 mL/L 85.0% H_3_PO_4_, 0.93 g/L CaSO_4_·2H_2_O, 18.2 g/L K_2_SO_4_, 14.9 g/L MgSO_4_·2H_2_O, 4.13 g/L KOH, 40.0 g/L glycerol), supplemented with 4.0 mL/L PTM1 (6.0 g/L CuSO_4_·5H_2_O, 0.088 g/L KI, 3.0 g/L MnSO_4_·H_2_O, 0.02 g/L H_3_BO_3_, 0.5 g/L CoCl_2_·6H_2_O, 20.0 g/L ZnCl_2_, 65.0 g/L FeSO_4_·7H_2_O, 0.2 g/L biotin, and 5 mL/L H_2_SO_4_). During the glycerol feeding phase, a 50.0% glycerol solution supplemented with 12 mL/L PTM1 was used. Pure methanol, supplemented with 12 mL/L PTM1, was employed as the inducer during the methanol induction phase. The fermentation conditions were as follows: an initial working volume of 3 L, a stirring speed set at 200 r/min, and an aeration volume at 1 vvm. The temperature during the growth phase was maintained at 30 °C, and the pH was adjusted to 5.5 using ammonia. The dissolved oxygen (DO) level was maintained above 25.0% through dissolved oxygen and speed coupling. After 24 h of cultivation, the incubation temperature was reduced to 20 °C. Methanol flow was initiated to induce expression, with flow addition synchronized with the DO level. Methanol flow addition was halted when the DO level fell below 25.0% and resumed when it rose above 25.0%. Samples were collected at 12-h intervals to measure the expression level and activity of PaDa-I-CD.

### Real-time fluorescence quantitative PCR

To determine the copy number of the target gene strains, genomic DNA was extracted using the FastDNA®SPIN Kit (MP Biomedicals, USA). The DNA concentration was determined using a NanodropTM (Thermo Fisher Scientific, USA) and standardized to 1 ng/µL using distilled water. The copy number was calculated using the described method (Aw and Polizzi 2016). The primers used for the real-time PCR are listed in Table S4. A total of 20 µL reaction mixture was prepared, including 10 µL SYRB® Green Realtime PCR Master, 1 µL DNA template, 0.6 µL primers (10 µmol/L), and 7.8 µL sterile water. The reaction conditions were as follows: incubation at 37 °C for 2 min, initial denaturation at 94 °C for 4 min, followed by 30 cycles of denaturation at 94 °C for 30 s, annealing at 58 °C for 30 s, and extension at 72 °C for 1 min. The final extension step was performed at 72 °C for 30 s, followed by a melting curve analysis to confirm the amplification specificity.

To measure the mRNA levels of PaDa-I-CD, BIP, ERO1, HAC1, and PDI, total RNA was extracted from strains using the yeast total RNA isolation kit from Shanghai Shenggong Biocompany (Shanghai, China). The PCR procedures followed the methods previously reported (Shen et al. 2020).

### Determination of the integration locus of the expression construct

To determine whether the expression construct specifically inserted into the AOX1 gene loci, primers Locus-AOX-F and Locus-PaDa-I-R were used to amplify the genome for each strain. If the expression construct was specifically inserted, the amplified product would be approximately 2000 bp. To determine whether the expression construct was specifically inserted into the HIS4 gene loci, primers Locus-HIS-F and Locus-pPIC9-R were used to amplify the genome. If the expression construct was specifically inserted, the amplified product would be approximately 2500 bp. The primers used to determine the integration locus are listed in Table S5. All PCR products were further sequenced by Beijing Qingke Biocompany to confirm the sequence.

### SDS-PAGE and Western blot analysis

The proteins secreted by the recombinant strains were analyzed using SDS-PAGE and Western blot techniques. SDS-PAGE was performed using a 10.0% polyacrylamide gel at 160 V for 1 h with the Bio-Rad cell system. The proteins from the gel were transferred to a nitrocellulose membrane using the dry transfer method. The membrane was then incubated at 37 °C for 1.5 h with 50 mM potassium phosphate buffer (PBS) containing 0.5% Tween 20. Next, the membrane was incubated with anti-6xHis polyclonal antibody (Shenggong, Shanghai, China) at a dilution of 1:5000 for 2 h. After washing, the membrane was further incubated with horseradish peroxidase-conjugated polyclonal anti-rabbit IgG (Shenggong, Shanghai, China) at a dilution of 1:15,000. Finally, the expression intensity of the target protein on the membrane was revealed using the ECL chemiluminescence method.

### Purification of PaDa-I-CD

The cultures from the bioreactor were centrifuged for 10 min at 8000 rpm to pellet the cells. HIS-select-nickel affinity gel column (Sigma-Aldrich, P003-NTA-µSphere, USA) was washed using 10 mM PBS (pH 7). Subsequently, the supernatant, equivalent to three times the column volume, was loaded onto the HIS-select-nickel affinity gel column. After allowing the supernatant to completely flow out, double the column volume of 10 mM imidazole buffer was loaded onto the column. Following that, 1 mL of 200 mM imidazole buffer was loaded onto the column, and the eluate was collected. The eluted protein solution was subjected to a desalting column (Tianyan Bio, A-55 mL, China) to replace the buffer system with 10 mM PBS (pH 7). Protein concentrations were assessed using the NanoOrange Protein Quantization Kit (Invitrogen).

### Determination of PaDa-I-CD activity

The peroxidative activity of PaDa-I-CD was determined using the oxidation of 2,2′-azino-bis(3-ethylbenzothiazoline-6-sulfonic acid) (ABTS) as the substrate. The activity assay was performed as previously reported methods with modification (Molina-Espeja et al. 2014). Briefly, the enzymatic reaction was initiated by adding 20 µL of purified enzyme solution (5 µg/µL) or culture supernatant to a solution containing 100 mM sodium phosphate/citrate buffer at pH 5.5, 0.3 mM ABTS, and 1 mM H_2_O_2_, resulting in a total volume of 200 µL. The unit enzyme activity was defined as the amount of enzyme required to oxidize 1 µmol of ABTS per minute.

### Synthesis of R-HPPA by the purified PaDa-I-CD

To biosynthesize R-HPPA, 200 µL of purified enzyme solution (5 µg/µL) was added to a solution containing 50 mM PBS (pH 7.5), 1 mM R-PPA, 1 mM H_2_O_2_, and 4 mM ascorbic acid, resulting in a total volume of 800 µL. The mixture was incubated at 30 °C for 1 h, and the reaction was terminated by adding 0.1 mL of 50.0% (wt/vol) trichloroacetic acid. The products were then analyzed by HPLC using a C18 column. The mobile phase comprised a mixture of acetonitrile and pH 2 phosphoric acid (in a ratio of 4:6), with a flow rate of 1.0 mL/min. Detection was performed at a wavelength of 220 nm, an injection volume of 5 µL, and the column temperature maintained at 30 °C.

Liquid chromatography/mass spectrometry (LC/MS) analyses were employed to further determine the molecular weight of the products, conducted using a reversed-phase Thermo Scientific Hypersil GOLD C18 column (dimensions: 100 × 2.1 mm, particle size: 3 μm). The mobile phase employed was an isocratic composition of 98.0% vol/vol methanol and 2.0% vol/vol acetonitrile. The column was operated at a temperature of 40 °C and a flow rate of 0.4 mL/min for a duration of 10 min. Electrospray ionization was performed in the negative ionization mode.

### Data processing

All experiments were performed in triplicate, and the results were presented as the mean ± SD. The data were plotted using Origin 8.1 software, and statistical analysis was conducted using one-way analysis of variance (ANOVA).

### Data availability

GenBank accession numbers for wild-type AaeUPO and UPO derived from *G. marginata* were B9W4V6 and KDR77412.1, respectively. GenBank accession numbers for chaperones BIP, ERO1, HAC1, and PDI were XM_002490982.1, CP014715.1, XP_002490039.1, and CAC33588.1, respectively.

## Results

### Effect of signal peptide on the secretion of PaDa-I-CD in *K. phaffii*

**Fig. 3:**
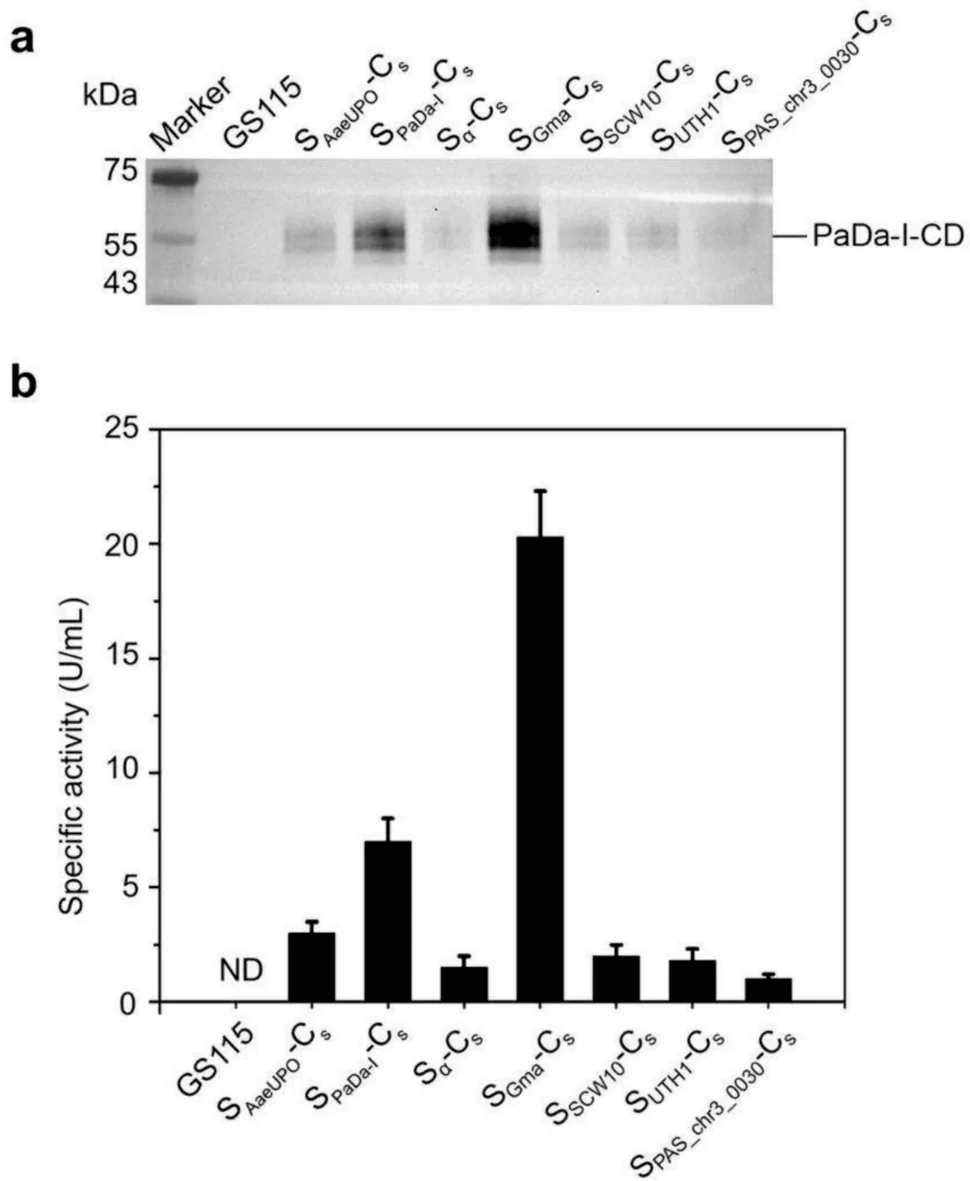
Effect of signal peptide on the secretion of PaDa-I-CD in *K. phaffii*. **a** The secretion levels of PaDa-I-CD in GS115, S_AaeUPO_-C_s_, S_PaDa−I_-C_s_, S_α_-C_s_, S_Gma_-C_s_, S_SCW10_-C_s_, S_UTH1_-C_s_, and S_PAS_chr3_0030_-C_s_. **b** The specific activities of ABTS for GS115, S_AaeUPO_-C_s_, S_PaDa−I_-C_s_, S_α_-C_s_, S_Gma_-C_s_, S_SCW10_-C_s_, S_UTH1_-C_s_, and S_PAS_chr3_0030_-C_s_. Representative single colonies were used for expression of PaDa-I-CD. There were 3 samples in each group. The supernatants of *K. phaffii* strain GS115 were used as the negative control

The impact of different signal peptides on the secretion of PaDa-I-CD was examined. To eliminate the impact of diverse integration locus on expression, we confirmed that the insertion of the PaDa-I-CD expression cassette in all strains occurred at the AOX1 loci through PCR and Sanger DNA sequencing (Fig. S1). The signal peptides used for comparison include the native signal peptide S_AaeUPO_, the evolved signal peptide S_PaDa−I_, and the recently reported signal peptide S_Gma_. Our group has identified several signal peptides with good secretion efficiency for many industrial enzymes (Shen et al. 2022), which were also used to compare their effects on the secretion of PaDa-I-CD. The Western blot analysis revealed that all engineered strains produced heterogeneous protein products with a molecular weight of around 55 kDa in the culture supernatant (Fig. 3a). In contrast, the parental strain *K. phaffii* GS115 did not show these bands. Consistent with the literature report (Molina-Espeja et al. 2014; Püllmann et al. 2021), the secretion efficiency of PaDa-I-CD followed the order of increase: S_AaeUPO_, S_PaDa−I_, S_Gma_. The effects of S_α_ and other signal peptides were less favorable. It is noteworthy that the detected molecular weight of the target protein was significantly larger than the theoretical molecular weight of PaDa-I-CD (35.95 kDa), and this phenomenon was independent of the signal peptide selection. Similar results have been observed by other groups, and the reason for this phenomenon was that PaDa-I-CD undergone glycosylation modification by the *K. phaffii* (Bormann et al. 2022a; Molina-Espeja et al. 2015). The enzyme activity assay using ABTS as a substrate was generally aligned with the Western blot analysis. The secretion efficiency of S_Gma_ was more than twice that of S_PaDa−I_ (Fig. 3b). Strains containing S_AaeUPO_ exhibited significantly higher enzymatic activity in the culture supernatant compared to the *K. phaffii* GS115, while strains with S_α_, S_SCW10_, S_UTH1_, and S_PAS_chr3_0030_ showed limited enzymatic activity.

### Enhancing PaDa-I-CD expression by increasing copy number of expression cassette

**Fig. 4:**
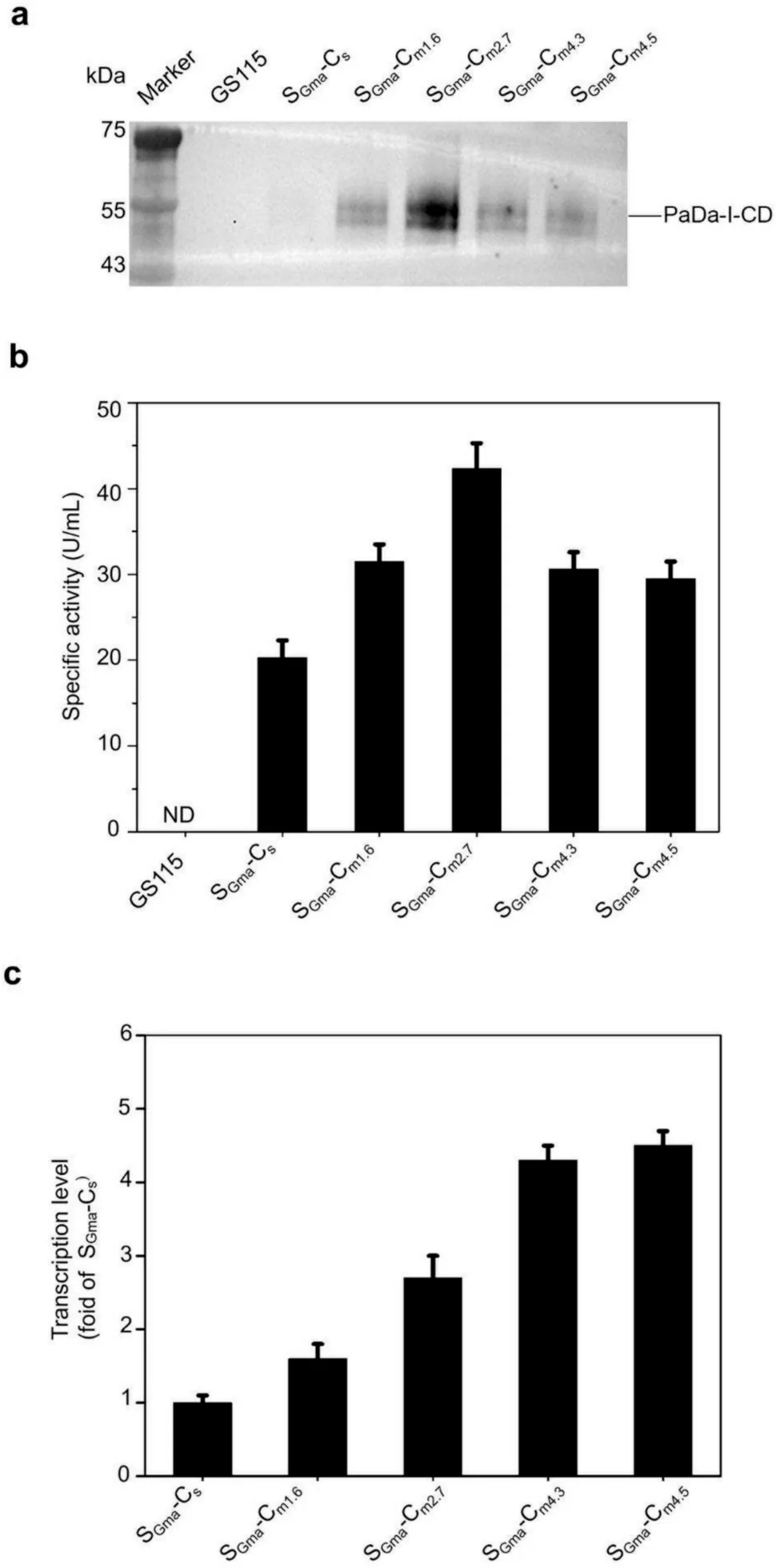
The expression and activity of PaDa-I-CD was increased by increasing the copy number. **a** The secretion levels of PaDa-I-CD in GS115, S_Gma_-C_s_, S_Gma_-C_m1.6_, S_Gma_-C_m2.7_, S_Gma_-C_m4.3_, and S_Gma_-C_m4.5_. **b** The specific activities of ABTS for GS115, S_Gma_-C_s_, S_Gma_-C_m1.6_, S_Gma_-C_m2.7_, S_Gma_-C_m4.3_, and S_Gma_-C_m4.5_. **c** The transcription levels of PaDa-I-CD in S_Gma_-C_s_, S_Gma_-C_m1.6_, S_Gma_-C_m2.7_, S_Gma_-C_m4.3_, and S_Gma_-C_m4.5_. Representative single colonies were used for expression of PaDa-I-CD. There were 3 samples in each group. The supernatants of *K. phaffii* strain GS115 were used as the negative control

Many studies have reported positive correlations between the expression levels of the target gene and the copy numbers of their expression cassette (Cai et al. 2019; Duman-Özdamar and Binay 2021; Ergün et al. 2021; YaPing et al. 2017; Zheng et al. 2019). To generate positive transformants with multiple copies of the expression cassette containing S_Gma_ and PaDa-I-CD, the linearized vector pPICZ-S_Gma_-PaDa-I-CD was introduced into *K. phaffii* GS115 through electroporation. Three days later, four transformants were observed on YPD plates supplemented with 800 µg/mL zeocin. The result of RT-PCR analysis indicated that the copy numbers of the expression cassette in these four transformants ranged from 1.6 to 4.5 (Table 1). The four transformants were cultured in shaking flasks for expression, and their supernatants were analyzed using Western blot (Fig. 4a). From a single copy to 2.7 copies, there was a significant increase in the expression level of PaDa-I-CD. However, when the copy number increased to 4.3 or more, the PaDa-I-CD expression level started to decline. The strain containing 2.7 copies was designated as S_Gma_-C_m2.7_ (Table 1). The enzyme activity assay using ABTS as a substrate also exhibited the same trend (Fig. 4b). Interestingly, the protein expression level of PaDa-I-CD was consistent with its transcriptional level (Fig. 4c). To exclude the potential impact of locus effect on expression levels in multicopy strains, we compared the strains containing expression construct integrated into the loci AOX1 and HIS4 (Fig. S2a and b). The Western blot analysis showed that the insertion of the expression construct into these two loci resulted in similar expression levels.

### Increasing the expression level of S_Gma_-C_m2.7_ through co-expressing chaperones

**Fig. 5:**
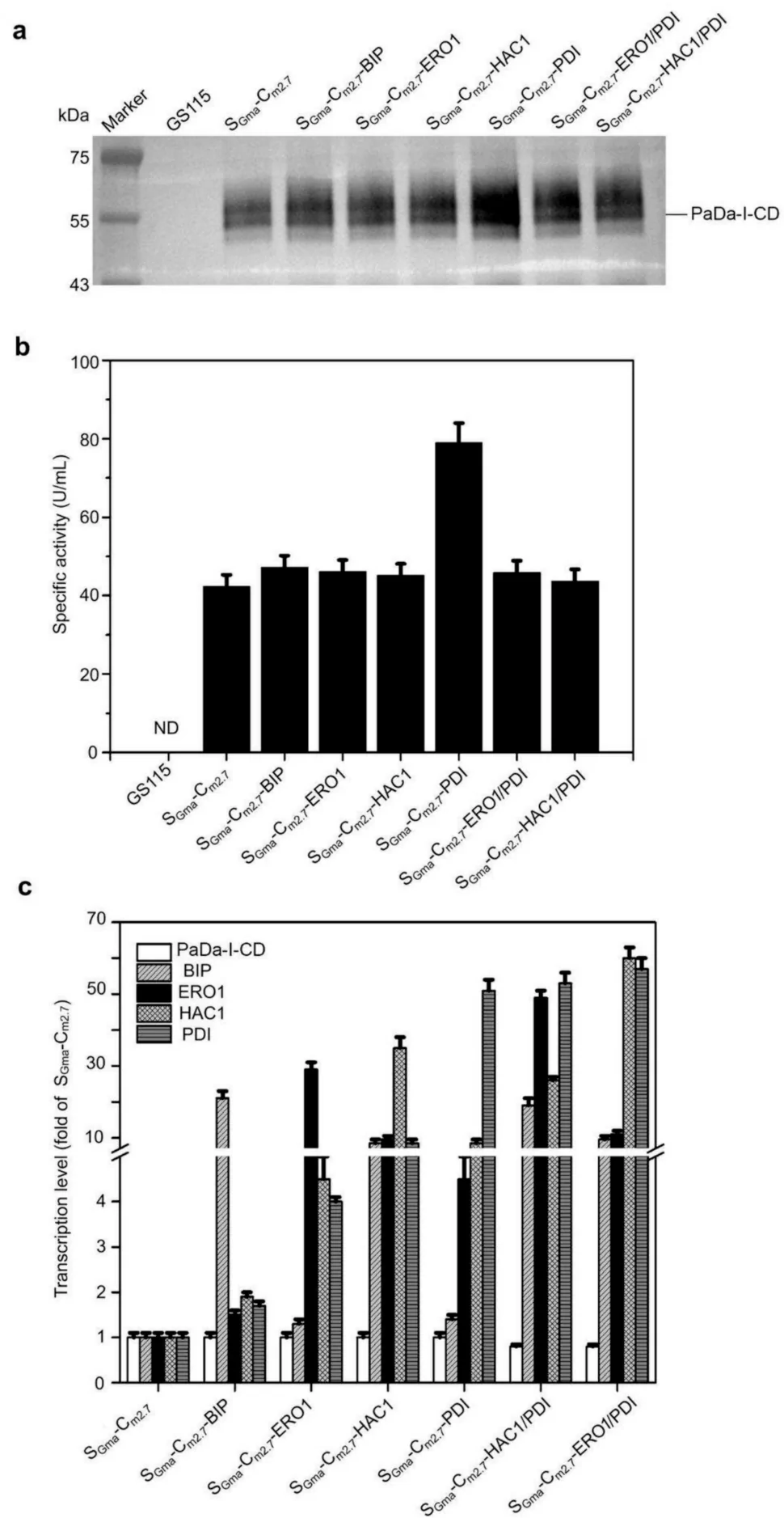
The expression and activity of PaDa-I-CD was increased by co-expression of PDI. **a** The secretion levels of PaDa-I-CD in GS115, S_Gma_-C_m2.7_, S_Gma_-C_m2.7_-BIP, S_Gma_-C_m2.7_-ERO1, S_Gma_-C_m2.7_-HAC1, S_Gma_-C_m2.7_-PDI, S_Gma_-C_m2.7_-ERO1/PDI, and S_Gma_-C_m2.7_-HAC1/PDI. **b** The specific activities of ABTS for GS115, S_Gma_-C_m2.7_, S_Gma_-C_m2.7_-BIP, S_Gma_-C_m2.7_-ERO1, S_Gma_-C_m2.7_-HAC1, S_Gma_-C_m2.7_-PDI, S_Gma_-C_m2.7_-ERO1/PDI, and S_Gma_-C_m2.7_-HAC1/PDI. **c** The transcription levels of PaDa-I-CD, BIP, ERO1, HAC1, and PDI in S_Gma_-C_m2.7_, S_Gma_-C_m2.7_-BIP, S_Gma_-C_m2.7_-ERO1, S_Gma_-C_m2.7_-HAC1, S_Gma_-C_m2.7_-PDI, S_Gma_-C_m2.7_-ERO1/PDI, and S_Gma_-C_m2.7_-HAC1/PDI. ND: not detected. There were 3 samples in each group. The supernatants of *K. phaffii* strain GS115 were used as the negative control

The secretion of PaDa-I-CD was significantly enhanced in the S_Gma_-C_m2.7_ compared to single-copy strains. However, it was well known that higher levels of protein synthesis in multicopy strains can lead to a relative insufficiency of chaperones involved in protein folding. Consequently, a large number of misfolded proteins may accumulate in the endoplasmic reticulum (ER), adversely affecting both the productivity of heterologous proteins and the normal metabolism of *K. phaffii*. To alleviate the metabolism burden on the S_Gma_-C_m2.7_, a series of chaperones were co-expressed in it, as depicted in Fig. 5a and b. The strain co-expressing PDI (S_Gma_-C_m2.7_-PDI) showed exhibited an approximately onefold increase in both the expression level and activity in its supernatant. Neither co-expressing BIP, ERO1, nor HAC1 had a significant impact on S_Gma_-C_m2.7_. Protein sequence analysis revealed the presence of three cysteine residues capable of forming disulfide bonds in PaDa-I, leading to hypothesize that the increased secretion of PaDa-I-CD through PDI co-expression was attributed to the role of PDI in facilitating disulfide bond formation and error correction (Benham et al. 2013). Previous studies have demonstrated the synergistic role of PDI with other molecular chaperones. Therefore, we investigated the co-expression of additional molecular chaperones, ERO1 or HAC1, on the foundation of S_Gma_-C_m2.7_-PDI. However, both Western blot analysis and the activity assay revealed no significant improvement (Fig. 5a and b).

The analysis of transcription levels showed that overexpressing molecular chaperones had no effect or even a reduction in the mRNA levels of PaDa-I-CD (Fig. 5c). Co-expression of individual chaperones had varying degrees of enhancement on the mRNA levels of other chaperones. Particularly in strains co-expressing HAC1 (S_Gma_-C_m2.7_-HAC1), the mRNA levels of BIP, ERO1, and PDI showed at least a tenfold increase. Interestingly, only in the host co-expressing PDI (S_Gma_-C_m2.7_-PDI), there was a significant increase in the expression level of PaDa-I-CD. These results indicated that the impact of overexpressing molecular chaperones on host metabolism and the expression of exogenous genes was highly complex (Shen et al. 2020).

### Optimizing expression conditions in shaking flasks

**Fig. 6:**
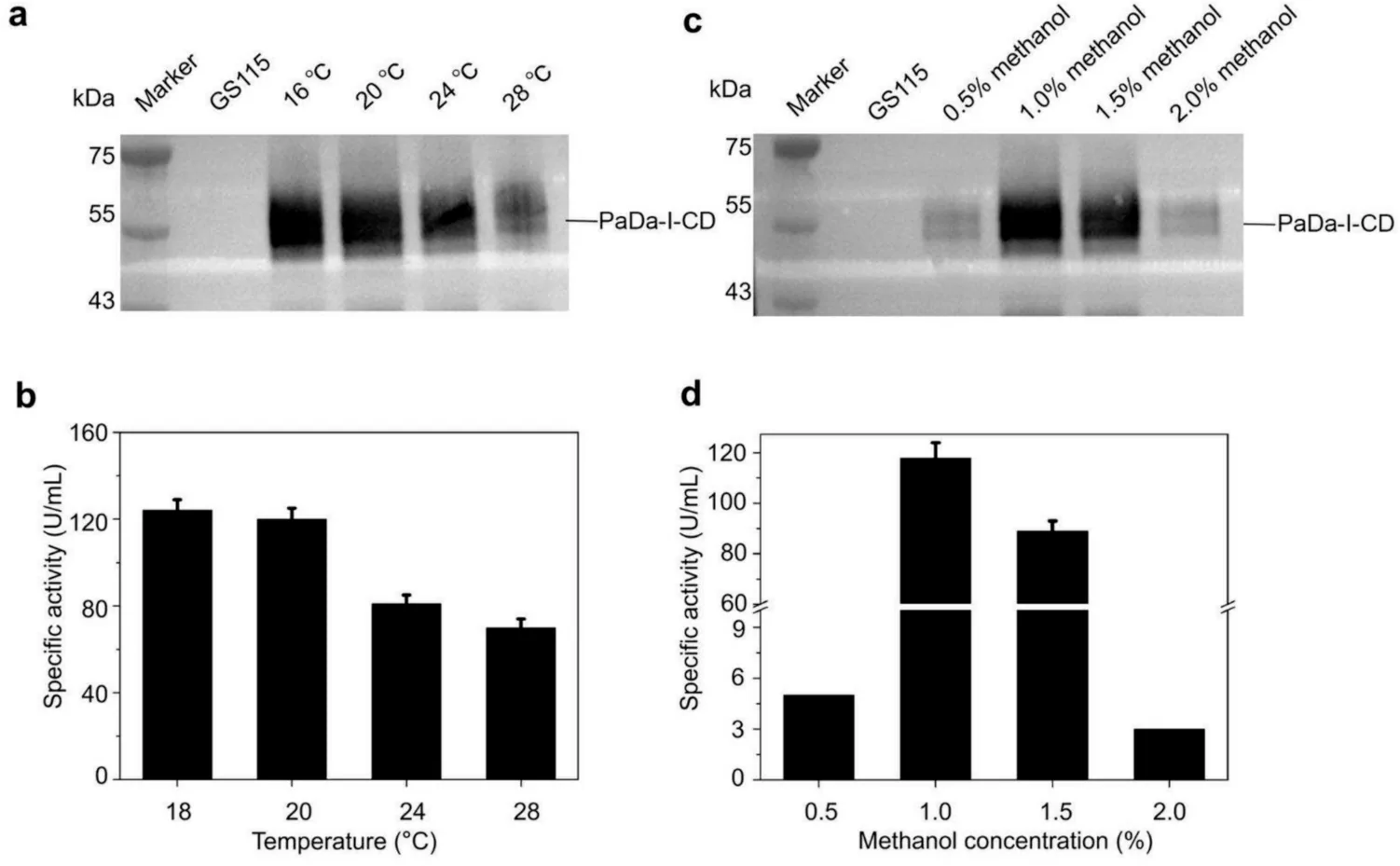
The effects of temperature and the amount of methanol induction concentration on expression and activity of PaDa-I-CD. **a** The secretion levels of PaDa-I-CD in S_Gma_-C_m2.7_-PDI at 16 ℃, 20 ℃, and 24 ℃, and 28 ℃. **b** The specific activities of ABTS for S_Gma_-C_m2.7_-PDI at 16 ℃, 20 ℃, 24 ℃, and 28 ℃. **c** The secretion levels of PaDa-I-CD in S_Gma_-C_m2.7_-PDI at 0.5%, 1.0%, 1.5%, and 2.0% methanol. **d** The specific activities of ABTS for S_Gma_-C_m2.7_-PDI at 0.5%, 1.0%, 1.5%, and 2.0% methanol. There were 3 samples in each group.The supernatants of *K. phaffii* strain GS115 were used as the negative control

To provide optimal conditions for high-density fermentation to produce PaDa-I-CD, we optimized induction temperature and methanol induction concentration under shaking flask conditions for the S_Gma_-C_m2.7_-PDI. Within the range of 16 to 28 °C, the expression level of PaDa-I-CD showed an inverse correlation with temperature (Fig. 6a and b). This phenomenon was likely related to the nature of PaDa-I-CD, considering that the optimal temperature of *K. phaffii* falls within the range of 28–30 °C. Considering that too low of an induction temperature was challenging to control for scale-up production and was also unfavorable for yeast growth, we chosen to further optimize induction conditions at 20 °C in subsequent experiments.

During the induction phase, methanol serves as both an inducer and the sole carbon source. Insufficient methanol can lead to inadequate induction, while excessive methanol can inhibit strain growth and metabolism. Thus, optimizing the amount of methanol added was crucial for enhancing the efficiency of PaDa-I-CD expression. The optimal methanol addition amount was determined to be 1.0% (Fig. 6c and d). When 1.5% methanol was used, a significant decrease in the level of expression was observed.

### High-density fermentation culture of recombinant *K. phaffii*

**Fig. 7:**
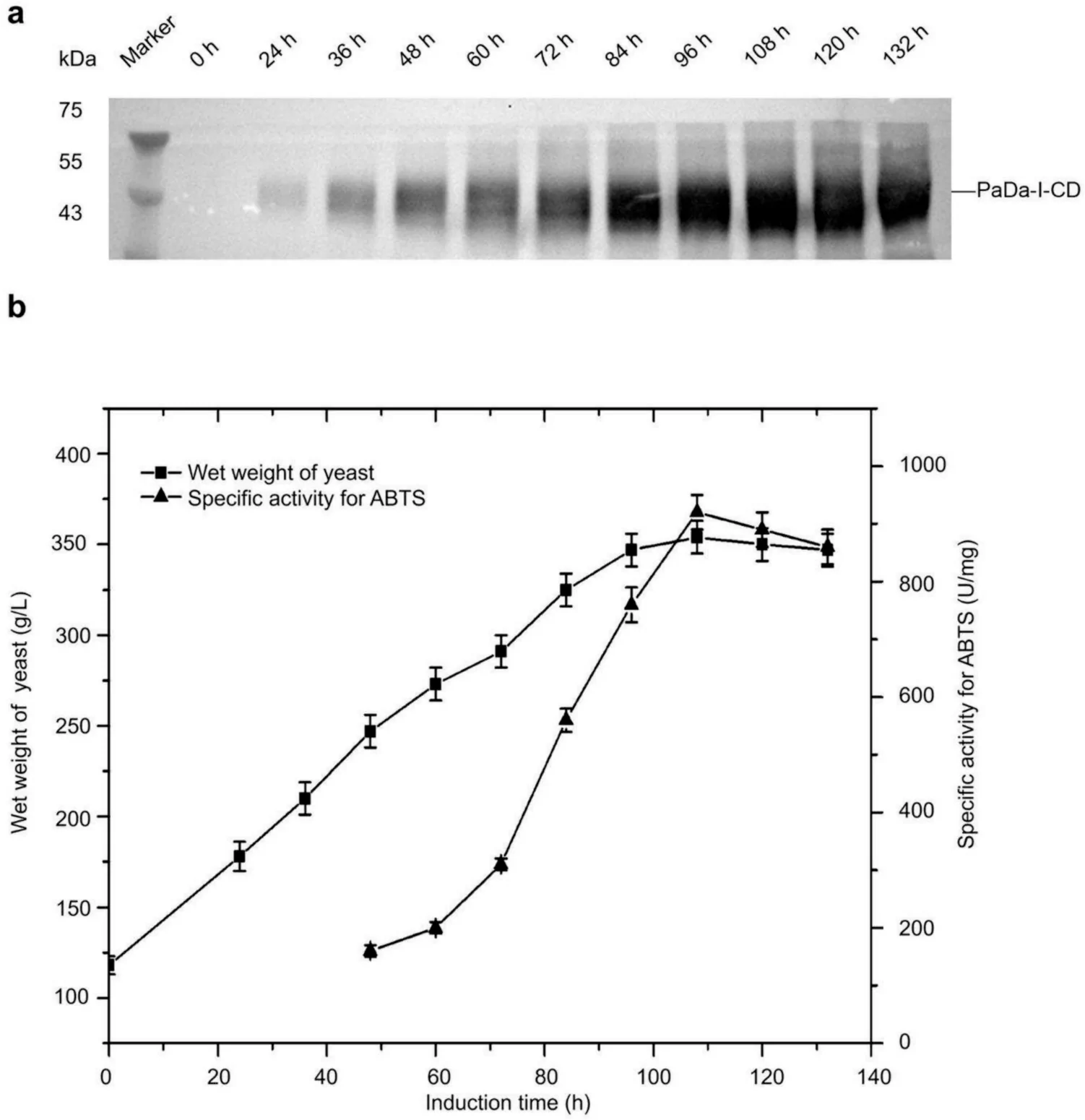
The production of PaDa-I-CD by high-density fermentation in 5 L bioreactor. **a** The PaDa-I-CD level at different induced time. **b** The wet weight of yeast and the specific activities of ABTS for samples collected at different induced time

We employed a 5-L bioreactor for high-density fermentation of the recombinant strain S_Gma_-C_m2.7_-PDI. The fermentation process was conducted at a temperature of 30 °C during the cell growth phase, with an induction temperature of 20 °C. The pH was maintained at 5.5 using 25.0% ammonia. Samples were collected every 12 h during the induction phase. The Western blot analysis and activity analysis showed that the expression of PaDa-I-CD can be observed as early as 24 h and 48 h post-induction, respectively (Fig. 7a and b). The expression level of PaDa-I-CD reached its maximum value at 108 h, after which it started to decline. Throughout the entire induction process, the biomass continued to increase.

### The biological transformation of R-PPA to R-HPPA using purified PaDa-I-CD

**Fig. 8:**
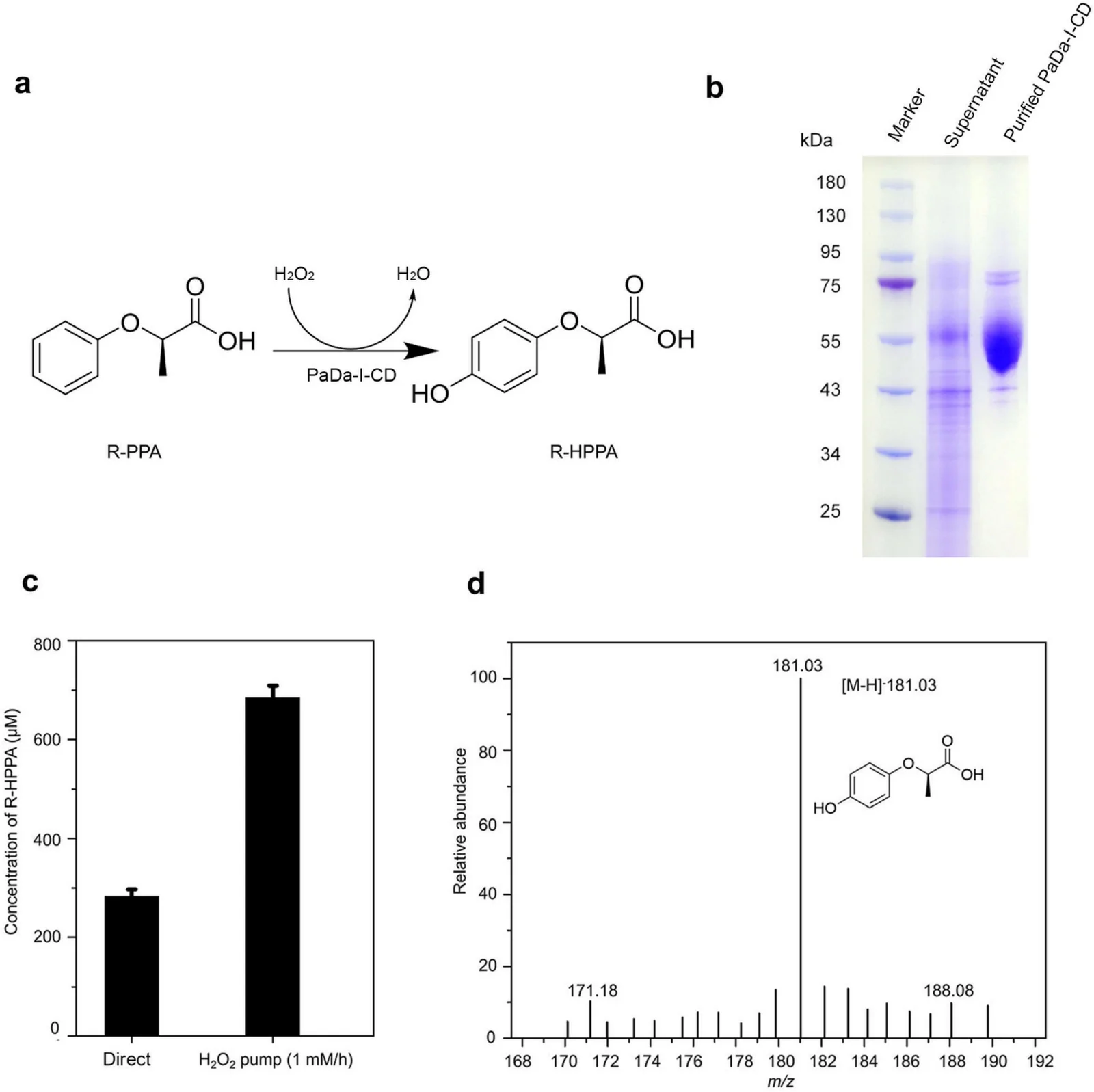
The biological transformation of R-PPA to R-HPPA using purified PaDa-I-CD. **a** Schematic of the reaction equation. **b** SDS-PAGE analysis of the fermentation supernatant and purified PaDa-I-CD. **c** The yields of R-HPPA. Direct, H_2_O_2_ added at the beginning of the reaction and is not supplemented later; H_2_O_2_ pump (1 mM/h): H_2_O_2_ was slowly added to the reaction system at a rate of 1 mM/h using a peristaltic pump, and the total reaction time is 2 h. **d** Mass spectra result of the product

In order to assess the industrial potential of PaDa-I-CD, we attempted to utilize purified enzyme for the synthesis of valuable compound R-HPPA from R-PPA (Fig. 8a and b). Ascorbic acid was introduced into the reaction system as a scavenger of free radicals to prevent excessive oxidation and polymerization of the products. In the first transformation system, hydrogen peroxide was added in a single dose, ultimately successfully generating approximately 284 µM of R-HPPA (Fig. 8c). Considering the potential toxicity of high concentrations of hydrogen peroxide to UPO (Romero et al. 2022), in the second transformation system, we introduced hydrogen peroxide using a continuous addition method, resulting in a final concentration of 684 µM of R-HPPA (Fig. 8c). The mass spectrum analysis of the product demonstrated a dominant [M-H]^−^ ion with an m/z of 181.03, which was consistent with the expected m/z for R-HPPA (Fig. 8d).

## Discussion

Fungal UPOs have garnered widespread attention since their initial discovery (Ullrich et al. 2004). However, efficiently expressing them in rapidly growing hosts has been a challenge, significantly hindering their industrial applications. UPOs can be phylogenetically sorted into two families based on their length (Hofrichter et al. 2020). Short-type UPOs are typically around 26 kDa in length, utilizing a histidine residue as a charge stabilizer. Long-type UPOs are generally around 44 kDa in length, using an arginine residue as a charge stabilizer. Current researches indicated that heterologous expression of long-type UPOs is more challenging compared to short-type UPOs. Efficient heterologous expression has only been reported for few long-type UPOs (Babot et al. 2013; Bormann et al. 2022b; Ebner et al. 2023; Molina-Espeja et al. 2014). The present study, using PaDa-I as a representative of long-type UPOs, investigated strategies to enhance its expression in *K. phaffii*.

Regarding the effectiveness of the secretion peptide, our results align with the literature report, showing that S_Gma_ exhibited very high efficiency (Püllmann et al. 2021). The authors of the above-mentioned paper proposed a hypothesis suggesting that, for the expression of PaDa-I, the closer the secretion peptide sequence and length are to its native secretion peptide, such as S_Gma_, the better the secretion efficiency. This pattern holed true in our experimental results, where attempted with four secretion peptides with very low homology to S_PaDa−I_ were unsuccessful (Fig. 3a and b).

Increasing the copy number of expression cassettes is a common method to enhance the expression of the target protein. We observed that 2.7 copies were optimal for PaDa-I-CD expression. Further, increasing copy numbers can negatively impact its expression (Fig. 4a and b). The potential reason behind this effect could be the metabolic stress imposed on *K. phaffii* due to the elevated copy numbers (Che et al. 2020).

The difficulty in heterologous expression of long-type UPOs compared to short-type UPOs is generally attributed to the more complex structure of long-type UPOs (Gomez de Santos et al. 2021; Püllmann and Weissenborn 2021). Surprisingly, to the best of our knowledge, there have been no study reporting the role of co-expressed chaperones on long-type UPOs. In the strain S_Gma_-C_m2.7_, overexpression of PDI increased PaDa-I-CD expression by approximately twofold, proving the crucial role of disulfide bond formation for PaDa-I-CD maturation.

## Supplementary Information

Below is the link to the electronic supplementary material.

Supplementary file1 (PDF 305 KB)
