# Supplementary material for: Enhancing the expression of the unspecific peroxygenase in Komagataella phaffii through a combination strategy
Source: Appl Microbiol Biotechnol. 2024 May 6;108(1):320. doi: 10.1007/s00253-024-13166-7 (PMC11074022; doi:10.1007/s00253-024-13166-7)
Supplement: Supplementary file 1 — Supplementary file1 (PDF 305 KB) [file 253_2024_13166_MOESM1_ESM.pdf]

Enhancing the expression of unspecific peroxygenase in *Komagataella phaffii* through a combination strategy

Li-Xiang Zhao[a,b], Shu-Ping Zou [a,b], Qi Shen[a,b], Ya-Ping Xue[a,b,*], Yu-Guo Zheng[a,b]

[a] Key Laboratory of Bioorganic Synthesis of Zhejiang Province, College of Biotechnology and Bioengineering, Zhejiang University of Technology, Hangzhou 310014, PR China

[b] Engineering Research Center of Bioconversion and Biopurification of Ministry of Education, Zhejiang University of Technology, Hangzhou 310014, PR China

*Corresponding author: Ya-Ping Xue, Key Laboratory of Bioorganic Synthesis of Zhejiang Province, College of Biotechnology and Bioengineering, Zhejiang University of Technology, Hangzhou, 310014, PR China.

E-mail address: xyp@zjut.edu.cn (Y.-P. Xue)

Table S1. The site-directed mutagenesis primers utilized in this study

primer	Primer sequence5′ →3′	Site-directed mutagenesis
FP-12	GGTTtttGCTGTTGGTGTTGTTGCTTTTCCAG	Y[12]F
RP-12	CACCAACAGCaaaAACCAAGGTTGGAAACAATGGG	
FP-1415	TTGCTgctcgtGTTGTTGCTTTTCCAGATTACGC	V[14]A and G[15]R
RP-1415	CAACAACacgagcAGCAAAAACCAAGGTTGGAAAC	
FP-21	CTTTTCCAgctTACGCTTCCTTGGCTGGTTTG	D[21]A
RP-21	AGCGTAagcTGGAAAAGCAACAACACCAACAG	

Table S2. Signal peptides used in this study

Signal peptide	Strains	Amino acid sequence	Nucleotide sequences
S_{AaeUPO}	*C. aegerita*	MKYFPLFPTLVF AARVVAFPAYA SLAGLSQQELD AIIPTLEAR	ATGAAGTATTTTCCATTGTTTCCAACTT TGGTTTTTGCTGCAAGAGTTGTGGCCTT TCCAGCTTACGCTAGCTTGGCTGGTTTG TCACAACAAGAATTAGATGCTATTATC CCAACCTTGGAAGCTAGA
$S_{PaDa\text{-}I}$	*C. aegerita*	MKYFPLFPTLV YAVGVVAFPDY ASLAGLSQQEL DAIIPTLEAR	ATGAAATATTTCCCATTGTTTCCAACTT TGGTTTACGCCGTCGGTGTTGTTGCTTT CCCTGATTACGCTTCATTGGCTGGTCTG TCTCAACAAGAATTGGATGCTATCATTC CAACATTAGAAGCTAGA
S_{α}	*S. cerevisiae*	MRFPSIFTAVLF AASSALAAPVN TTTEDETAQIPA EAVIGYSDLEGD FDVAVLPFSNST NNGLLFINTTIA SIAAKEEGVSLE KREAEA	ATGAGGTTTCCATCCATTTTTACAGCTG TTTTATTTGCCGCCTCTAGTGCTTTGGC TGCACCAGTTAACACTACTACTGAAGA TGAAACAGCCCAAATTCCAGCTGAAGC TGTGATTGGCTATTCGGATTTGGAAGGT GATTTTGATGTTGCTGTTTTGCCATTTTC TAATTCTACAAATAACGGTTTGTTGTTC ATTAACACTACCATTGCCTCTATCGCTG CAAAAGAAGAAGGTGTTTCCCTGGAGA AAAGAGAAGCTGAAGCC
S_{Gma}	*G. marginata*	MRGTPIFASLIA LFAHAAIAFPAY GSLAGLTREQL DEILPTLEIRA	ATGAGAGGTACTCCAATTTTTGCTTCCT TGATTGCATTGTTTGCTCATGCAGCTAT CGCTTTTCCAGCATATGGTTCTTTGGCC GGCTTGACTAGAGAACAATTGGACGAA ATTTTGCCAACATTGGAAATTAGAGCT
S_{SCW10}	*K. phaffii*	MQVKSIVNLLL ACSLAVARPLE HAHHQHDKRG	ATGCAAGTCAAGTCTATTGTTAACTTGT TGTTGGCTTGTTCTTTGGCTGTTGCTAG ACCATTGGAACATGCTCACCATCAACA

		VWWVTKTIVVD GSTVEATAAAQ VQEHAE	TGATAAGAGAGGTGTTTGGTGGGTTAC AAAGACAATTGTTGTTGATGGTTCTACT GTCGAAGCTACAGCTGCTGCTCAAGTT CAAGAACACGCAGAA
S_{UTH1}	*K. phaffii*	MKSQLIFMALA SLVASAPLEHQ QQHHKHEKRAV VTQTVTVAAGQ TAAAGSAQAWT SSAA	ATGAAATCACAATTGATTTTTATGGCTT TGGCTTCTTTAGTTGCTTCTGCACCATT AGAACATCAACAACAACATCATAAGCA TGAAAAGAGAGCAGTTGTTACTCAAAC TGTTACTGTTGCTGCTGGTCAAACTGCA GCTGCTGGTTCTGCTCAAGCTTGGACTT CTTCAGCTGCA
$S_{PAS\_chr3\_0030}$	*K. phaffii*	MKFAISTLLILQ AAAVFAAFPISD ITWVSERTDAST AYLSDWFWVSF VFSTAGSDETIA GDATI	ATGAAGTTTGCTATTTCTACTTTGTTGA TCTTGCAAGCCGCTGCAGTTTTTGCTGC ATTTCCTATCTCTGACATTACTTGGGTT TCAGAAAGAACTGATGCCTCAACAGCC TACTTATCTGATTGGTTTTGGGTTTCCTT CGTTTTTAGCACTGCCGGTTCTGATGAA ACTATTGCTGGTGATGCTACTATT

Table S3. Primers used for the construction of plasmids

Primers	Primer sequence (5'-3')	Amplified fragment
FP-pPICZ-PaDa-I-CD	GAACCTGGTTTGCCACCTGG	linearized pPICZ-PaDa-I-CD
RP-pPICZ-PaDa-I-CD	ATCTCTACCGTATGGAAAAACTTGAGT	
FP-S_{α}	tttccatacggtagagatTGAGTTTGTAGCCTTAGACATGACTGTTC	S_{α} gene
RP-S_{α}	aggtggcaaaccaggttcAGCTTCAGCCTCTCTTTTCTCG	
FP-S_{Gma}	tttccatacggtagagatTGAGTTTGTAGCCTTAGACATGACTGTTC	S_{Gma} gene
RP-S_{Gma}	aggtggcaaaccaggttcAGCTCTAATTTCCAAGGTAGGCAAAAT	
FP-S_{SCW10}	tttccatacggtagagatTGAGTTTGTAGCCTTAGACATGACTGTTC	S_{SCW10} gene
RP-S_{SCW10}	aggtggcaaaccaggttcTTTGGTTCCAGCGGAGAAGC	
FP-S_{UTH1}	tttccatacggtagagatTGAGTTTGTAGCCTTAGACATGACTGTTC	S_{UTH1} gene
RP-S_{UTH1}	aggtggcaaaccaggttcCTTGGAGTAGCCGGCTTCAC	
FP-$S_{PAS\_chr3\_0030}$	tttccatacggtagagatTGAGTTTGTAGCCTTAGACATGACTGTTC	$S_{PAS\_chr3\_0030}$ gene
RP-$S_{PAS\_chr3\_0030}$	aggtggcaaaccaggttcTTTGAGTGTAAAGAAAACAACTCCTTCAAG	

FP-BIP	ATGCTGTCGTTAAAACCATCTTGG	
RP-BIP	CTACAACTCATCATGATCATAGTCATAGTCGT	BIP gene
FP-pPIC3.5K-BIP	gatcatgatgagttgtagGCGGCCGCGAATTAATTCGC	
RP-pPIC3.5K-BIP	tggttttaacgacagcatGAATTCTACGTAGGATCCTTCGAATAATTA	Linearized pPIC3.5K-BIP
FP-ERO1	ATGAGGATAGTAAGGAGCGTAGC	
RP-ERO1	TTACAAGTCTACTCTATATGTGGTATCTCGG	ERO1 gene
FP-pAO815-ERO1	tatagagtagacttgtaaTCAAGAGGATGTCAGAATGCCAT	Linearized pAO815-ERO1
RP-pAO815-ERO1	gctccttactatcctcatCGTTTCGAATAATTAGTTGTTTTTTGATC	
FP-PDI	ATGCAATTCAACTGGGATATTAAAACTG	
RP-PDI	TTAAAGCTCGTCGTGAGCGTCTG	PDI gene
FP-pPIC3.5K-PDI	gctcacgacgagctttaaGCGGCCGCGAATTAATTCGC	
RP-pPIC3.5K-PDI	atcccagttgaattgcatGAATTCTACGTAGGATCCTTCGAATAATTA	Linearized pPIC3.5K-PDI
FP-HAC1	ATGCCCGTAGATTCTTCTCATAAGACA	HAC1 gene
RP-HAC1	TCACCTGATCGCTATGCATG	
FP-pAO815-HAC1	tgcatagcgatcaggtgaTCAAGAGGATGTCAGAATGCCAT	Linearized pAO815-HAC1
RP-pAO815-HAC1	agaagaatctacgggcatCGTTTCGAATAATTAGTTGTTTTTTGATC	
FP-PDI-cassette	GCAGATCGGGAACACTGAAAAAT	PDI-cassette gene
RP-PDI-cassette	ATCGATAAGCTTGCACAAACGAA	
FP-pAO815-ERO1-PDI	ttgtgcaagcttatcgatGTTTATCACAGTTAAATTGCTAACGCAG	Linearized pAO815-ERO1
RP-pAO815-ERO1-PDI	atttttcagtgttcccgaGCATTAGGATCCGCACAAAC	
FP-pAO815-HAC1-PDI	ttgtgcaagcttatcgatGTTTATCACAGTTAAATTGCTAACGCAG	Linearized pAO815-HAC1
RP-pAO815-HAC1-PDI	atttttcagtgttcccgaGCATTAGGATCCGCACAAAC	
S_{Gma}-PaDa-I-CD-F	caactaattattcgaaacgATGAGAGGTACTCCAATTTTCGCTT	S_{Gma}-PaDa-I-CD fragment
S_{Gma}-PaDa-I-CD-R	TTAGTGATGATGATGATGGTGATCTCTATTAGTGATGATGATGATGGTGATCTCTA	
pPIC9-F	TGTTCCTCAGTTCAAGTTGGGCAC	Linearized pPIC9
pPIC9-R	TTCGAATAATTAGTTGTTTTTTGATCTTCTCAAGT	

Table S4. Primers used for RT-PCR

Primer	Primer sequence (5'-3')
RT FP-PaDa-I-CD	CGAACATGGTACTTTTGAGGGTGAT
RT RP-PaDa-I-CD	CGTAAGCGGTGAAGAATCTGAAATC
RT FP-GAP	GGTATTAACGGTTTCGGACGTATTG
RT RP-GAP	GATGTTGACAGGGTCTCTCTCTTGG
RT FP-BIP	ACAGAGTTGTTCGCCACTTCGTT
RT RP-BIP	ACCGTCGACGAAAGAGTCAATCTCA
RT FP-ERO1	CTCAAGGAGCACAGGGTATT
RT RP-ERO1	ATCTAGTCACGTTGCGGAAT
RT FP-PDI	ACCACATTTTACGGAGTTGCCGGT
RT RP-PDI	CCTCGCCAGGTCTGACAAGCA
RT FP-HAC1	AGTTACCCAGGAAAATGAAAGTCTTAAAC
RT RP-HAC1	CTGAATCCTCCGGAAGACTCAAAG

Table S5. The primers used to determine the integration locus

Primer	Primer sequence (5'-3')
Locus-AOX-F	GGGAGTGGAGGAGTAAATGAAATGT
Locus-PaDa-I-R	CATGGATGAGCTTCATCGTTAACC
Locus-HIS-F	GACTCACTGATAATAAAAATACGGCTTC
Locus-pPIC9-R	TGTGGATCTATCGAATCTAAATGTAAG

Fig S1

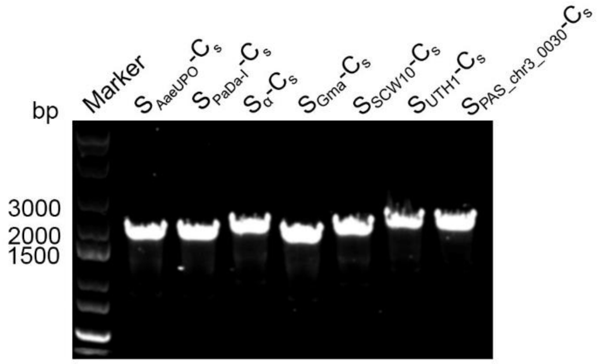

Fig. S1 Determination of the integration locus by PCR. Primers Locus-AOX-F and Locus-PaDa-I-R were utilized to amplify the genome for each strain.

Fig. S2

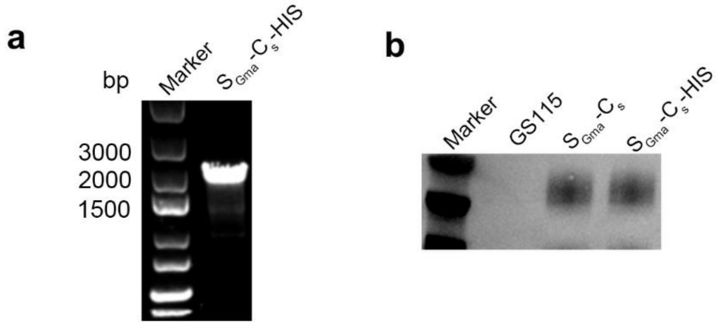

Fig. S2 The insertion of the expression construct into loci AOX1 and HIS4 resulted in similar expression levels. **a** Determination of the integration locus by PCR. The strain with expression construct integrated into HIS4 loci was designated as S_{Gma}-C_s-HIS. Primers Locus-HIS-F and Locus-pPIC9-R were utilized to amplify the genome for S_{Gma}-C_s-HIS. **b** The secretion levels of PaDa-I-CD in S_{Gma}-C_s and S_{Gma}-C_s-HIS. The supernatants of *K. phaffii* strain GS115 were used as the negative control.
